# Mapping and quantification of ferruginous outcrop savannas in the Brazilian Amazon: A challenge for biodiversity conservation

**DOI:** 10.1371/journal.pone.0211095

**Published:** 2019-01-17

**Authors:** Pedro Walfir M. Souza-Filho, Tereza C. Giannini, Rodolfo Jaffé, Ana M. Giulietti, Diogo C. Santos, Wilson R. Nascimento, José Tasso F. Guimarães, Marlene F. Costa, Vera L. Imperatriz- Fonseca, José O. Siqueira

**Affiliations:** 1 Instituto Tecnológico Vale, Belém, Pará, Brazil; 2 Geosciences Institute, Universidade Federal do Pará, Belém, Pará, Brazil; 3 Gerência de Meio Ambiente–Minas de Carajás, Departamento de Ferrosos Norte, Vale S.A. Parauapebas, Pará, Brazil; Centre National de la Recherche Scientifique, FRANCE

## Abstract

The eastern Brazilian Amazon contains many isolated ferruginous savanna ecosystem patches (locally known as ‘canga vegetation’) located on ironstone rocky outcrops on the top of plateaus and ridges, surrounded by tropical rainforests. In the Carajás Mineral Province (CMP), these outcrops contain large iron ore reserves that have been exploited by opencast mining since the 1980s. The canga vegetation is particularly impacted by mining, since the iron ores that occur are associated with this type of vegetation and currently, little is known regarding the extent of canga vegetation patches before mining activities began. This information is important for quantifying the impact of mining, in addition to helping plan conservation programmes. Here, land cover changes of the Canga area in the CMP are evaluated by estimating the pre-mining area of canga patches and comparing it to the actual extent of canga patches. We mapped canga vegetation using geographic object-based image analysis (GEOBIA) from 1973 Landsat-1 MSS, 1984 and 2001 Landsat-5 TM, and 2016 Landsat-8 OLI images, and found that canga vegetation originally occupied an area of 144.2 km^2^ before mining exploitation. By 2016, 19.6% of the canga area was lost in the CMP due to conversion to other land-use types (mining areas, pasturelands). In the Carajás National Forest (CNF), located within the CMP, the original canga vegetation covered 105.2 km^2^ (2.55% of the CNF total area), and in 2016, canga vegetation occupied an area of 77.2 km^2^ (1.87%). Therefore, after more than three decades of mineral exploitation, less than 20% of the total canga area was lost. Currently, 21% of the canga area in the CMP is protected by the Campos Ferruginosos National Park. By documenting the initial extent of canga vegetation in the eastern Amazon and the extent to which it has been lost due to mining operations, the results of this work are the first step towards conserving this ecosystem.

## Introduction

**Fig 1:**
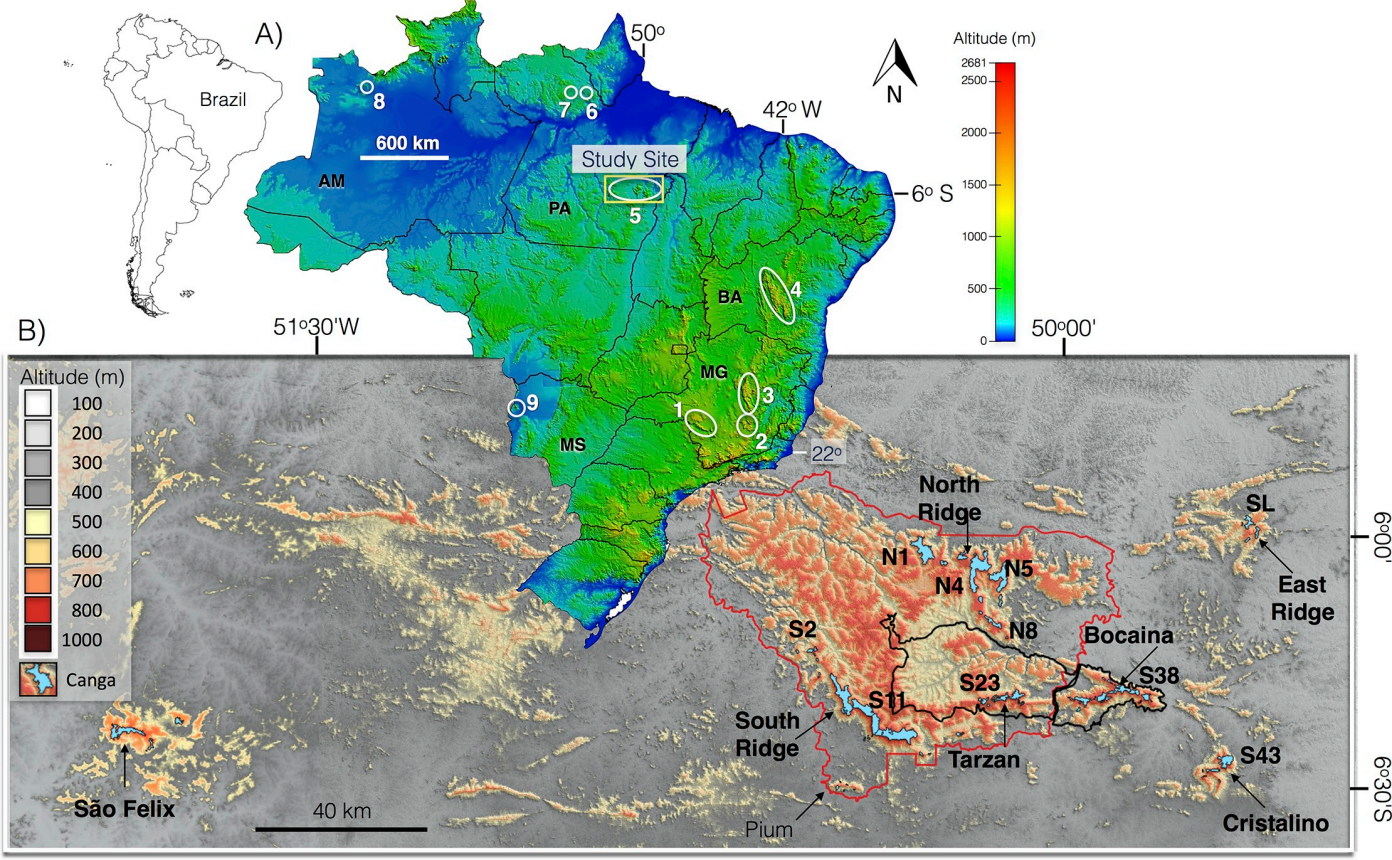
**A) Distribution of the main ferruginous outcrop savanna ecosystems (locally known as canga) in Brazil.** In Minas Gerais State (MG), canga vegetation occurs in Serra da Canastra (1), the Iron Quadrangle (2), and Serra do Cipó (3). In Bahia State (BA), the vegetation occurs in Chapada Diamantina (4). In Pará State (PA), the vegetation occurs in Serra dos Carajás (5), Maraconaí (6), and Maicuru (7). In Amazonas State (AM), the vegetation occurs in Serra dos Seis Lagos [24], while in Mato Grosso do Sul (MS), the vegetation occurs in Morraria de Urucum (9). N1, N4, N5, N8, S2, S11, S23, S38, and S43 are examples of geomorphic units located in the study area. B) **The Shuttle Radar Topography Mission (SRTM) elevation map of the study area with canga vegetation before mining implementation**. The red and black lines represent the boundaries of the Carajás National Forest (CNF) and the Campos Ferruginosos National Park (CFNP) protected areas, respectively. The digital elevation model (SRTM, 1 arc-second) was obtained from USGS Earth Explorer (https://earthexplorer.usgs.gov) and the CNF and CFNP shapefiles from ICMBIO (http://mapas.icmbio.gov.br/i3geo/datadownload.htm). All other layers and photos were produced by the authors and are copyright-free.

Several studies have investigated conservation and threats to biodiversity and ecosystem services in tropical rainforests [1]. Deforestation rates in the Amazon, the largest remaining tropical forest in the world, have also been well studied [2]. However, little information is available regarding the unique ecosystems found on ironstone rocky outcrops on the tops of plateaus and ridges. In the Carajás Mineral Province (CMP), located in the Eastern Amazon, these ferruginous outcrop savanna ecosystems are called “canga” [3] and occur within a dense forest matrix typical of the Amazon rainforest biome [4]. Canga vegetation, also associated with the presence of iron ore, is known to exist in at least two more regions in Brazil, namely, the Quadrilátero Ferrífero, or Iron Quadrangle [5], and the lateritic banks at Corumbá [6]. There are other types of open vegetation in the Amazon (Fig 1), but they are different from canga vegetation and are determined by different soil conditions (lateritic or very poor sandy soils). In 1967, geologists from United States Steel discovered these ferruginous outcrops on top of the ridges of the CMP, which is one of the most important metallogenic provinces in the world that contains large deposits of iron, as well as manganese, nickel, copper and gold [7]. Significant investments in mineral and ore exploration and exploitation have occurred over the past four decades [8]. Brazil’s constitution and National Forest Code require that in order to obtain a mining license, no net loss of biodiversity and only minimal environmental impacts can occur. Licensing processes demand basic information about biota and environmental services associated with future mining areas [9, 10]. Mining activities must be conducted in the interest of controlling their interference in the environment. Hence, it is necessary to present a Degraded Area Recovery Plan (PRAD in Portuguese), when the environmental viability of the project is assessed [11].

Compliance with legal demands brought opportunities for research, which has contributed to increasing the knowledge about flora in canga of the CMP [4] and in other Brazilian mining sites located in Minas Gerais, Bahia, and Mato Grosso do Sul (Fig 1). However, it is clear that there are few floristic links between the Amazonian canga and the species found in the Brazilian cerrado, which are prevalent in the Amazonian savanna in lowlands, sandy soils, or on top of plateaus, as seen between Venezuela and Suriname [12]. Two locations within our study area, the cangas of the Carajás National Forest (CNF) and the Campos Ferruginosos National Park (CFNP), contain 856 seed plant species, most of which are herbs (40%), with 24 endemic species. For invasive plant species, the same two localities contain 17 exotic invasive plant species, most of them located in the recently created CFNP [12]. However, knowledge of plant growth strategies and other factors that could affect the dynamics of recovery or rehabilitation of canga vegetation is still very limited [13]. Canga plateaus surrounded by evergreen forests are considered isolated entities, although little is known about the dispersal between plateaus. Recent genetic analyses have demonstrated that two perennial morning glories (*Ipomoea* spp.) exhibited gene flow between these canga plateaus, and genetic diversity in these species was not influenced by the size of the plateaus [14]. Another work focusing on obligate cave dwellers revealed decreasing community similarity with increasing distance between the caves, suggesting that these organisms are indeed moving between caves and plateaus [15].

As opposed to the suppression of canga vegetation from mining in the Iron Quadrangle (Minas Gerais State, southeastern Brazil), which began in the 18^th^ century, the suppression of canga vegetation in the CMP began only recently, in the 1980s. Some authors have described the environmental degradation of canga vegetation in Brazil [16, 17] and of a similar vegetation type in the ironstone ranges of Australia [18, 19], recommending the establishment of protected areas to guarantee their conservation. In the CMP, seven protected areas are in place, pursuing a balance between mining and conservation. On one hand, protected areas safeguard mining-licensed operations from illegal activities through a green protected belt; on the other hand, the mining companies participate in the protection of natural areas, preventing fires and undesired human occupation through regular surveillance [20]. This kind of protection appears to have been achieved inside protected areas of the Carajás region, where the forests are mostly undisturbed. In contrast, the surrounding areas of Carajás (the Itacaiúnas River watershed area) have lost 70% of its natural land cover (forests) over the past 40 years due to agriculture and cattle grazing [21]. The future expansion of mining is regulated by the CNF Management Plan that was recently published, which recommends that mining could expand until reaching 14% of the total CNF area [22]. However, the CNF Management Plan does not specify the minimum extent of canga that must be preserved. Hence, the current loss of canga areas is still a challenge since the areas of loss have only been estimated by analogic aerial photographs [23] that are not sufficiently accurate, unlike the orthorectified satellite images used in this study.

The accurate mapping and quantifying of canga vegetation areas within the eastern Amazon could be a first step to guide conservation strategies. Other authors have already discussed the importance of conserving the canga ecosystem and its rich biodiversity [12]. Human disturbances are a major threat and began in the 18^th^ century with vegetation suppression and anthropogenic burning in support of early mining activities, cattle industry, eucalyptus plantation and wood extraction [17]. Currently, the harvesting of ornamental plants (such as orchids), road construction, urbanization, and invasive species are also considered to be important threats [17].

In this study, we aim to evaluate the land-cover and land-use (LCLU) changes in the canga vegetation of the CMP (eastern Brazilian Amazon) during the cycle of mining operations in order to quantify the impact of mining on canga vegetation. The objectives of this study are (1) to present a geographical object-based Landsat image classification to quantitatively assess the extent of canga areas in the study area before mining projects were implemented in 1973; (2) to determine the extent of canga and forest areas around the beginning of mining activities (1984) and different snapshots in time afterwards, specifically in 2001 and 2016; and (3) to assess the average rate of canga vegetation suppression by land-use changes from one snapshot in time to another. This study is important due to the current lack of effective mapping and quantifying of changes to canga vegetation area using orthorectified satellite images during mining operations. This gap in our current knowledge is a challenge that hinders the national and local understanding of canga vegetation in a setting of open pit mining inside protected areas, as well as determining the necessary next steps to protect this vegetation.

## Materials and methods

This project was carried out in the Carajás National Forest under permission of IBAMA (SISBIO 35594–2).

### Study area

The study site is represented by the CMP ridges in the eastern Brazilian Amazon [7]. This region is recognized as a major Neoarchean tectonic province of the Amazonian Craton [25]. Geologically, this region is called the Carajás Formation, and it is composed of banded iron formations (BIFs) represented by jaspilites, with mafic rocks situated above and beneath it. Andesites, basalts, volcanoclastic materials, and gabbro are also present [26].

During the formation of these iron-rich deposits, the weathering processes of the Carajás Formation rocks occurred under humid climate conditions that allowed the formation of an extensively weathered profile on basic volcanic and BIF rocks. This alteration mantle contains iron-aluminous laterite, haematitic breccia, and ortho- and para-conglomerates [27], and acts as a surface crust on the tops of some ridges regionally represented by the Carajás Ridge [28].

The climate in the region is classified as the Aw type according to Köppen [29]. The region experiences high annual rainfall (~2,000 mm). Peak precipitation occurs during the rainy season between January and March, while the driest season occurs between June and August. Monthly temperatures vary between 25°C and 26°C, with the absolute minimum temperature between 16°C and 18°C between July and October, and the maximum temperature between 34°C and 38°C during all other months [30].

In the CMP, canga vegetation occurs over laterites and haematite breccia and conglomerates on top of some ridges with altitudes that range from 280 m to 904 m and average 670 m (Fig 1). In this paper, we subdivided the study site into seven geomorphic units: North (N1-N9), East (L1-L3), South (S1-S17), Tarzan (S18-S28), Bocaina (S29-S40), Cristalino (S43-S45), and Pium and São Felix (SF1-SF3) ridges. The nomenclature uses the letters N, S, L and SF to indicate North (*“Norte”*), South (*“Sul”*), East (*“Leste”*) and São Felix ridges [31], respectively (Fig 1). Mining projects in the CMP began in 1984 with the implantation of the N4-N5 mines. Later, the East Ridge and S11D mines began operating in 2012 and 2016, respectively [32]. It is important to emphasize that the largest iron ore mines N4-N5 and S11D occur inside the CNF. Only the East ridge mine is located outside of the CNF protected area.

### Remote sensing dataset, digital image processing and field data collection

Four Landsat images were used in this study. The 1973 Landsat-1 MMS image, with an 80 m spatial resolution, was only used to provide a visual observation of the canga areas before the first cycle of Amazon settlement. The 1984 and 2001 Landsat-5 TM and 2016 Landsat-8 OLI images were acquired in the Level 1 Terrain (L1T) format. The images were orthorectified with 30 m pixels to the Universal Transverse Mercator (UTM) 22S zone projection and datum WGS84. All 1984, 2001 and 2016 Landsat images were converted to ground reflectance in percentages. For each Landsat image, we derived the Normalized Difference Vegetation Index (NDVI) between vegetated areas and exposed soil [33].

**Fig 2:**
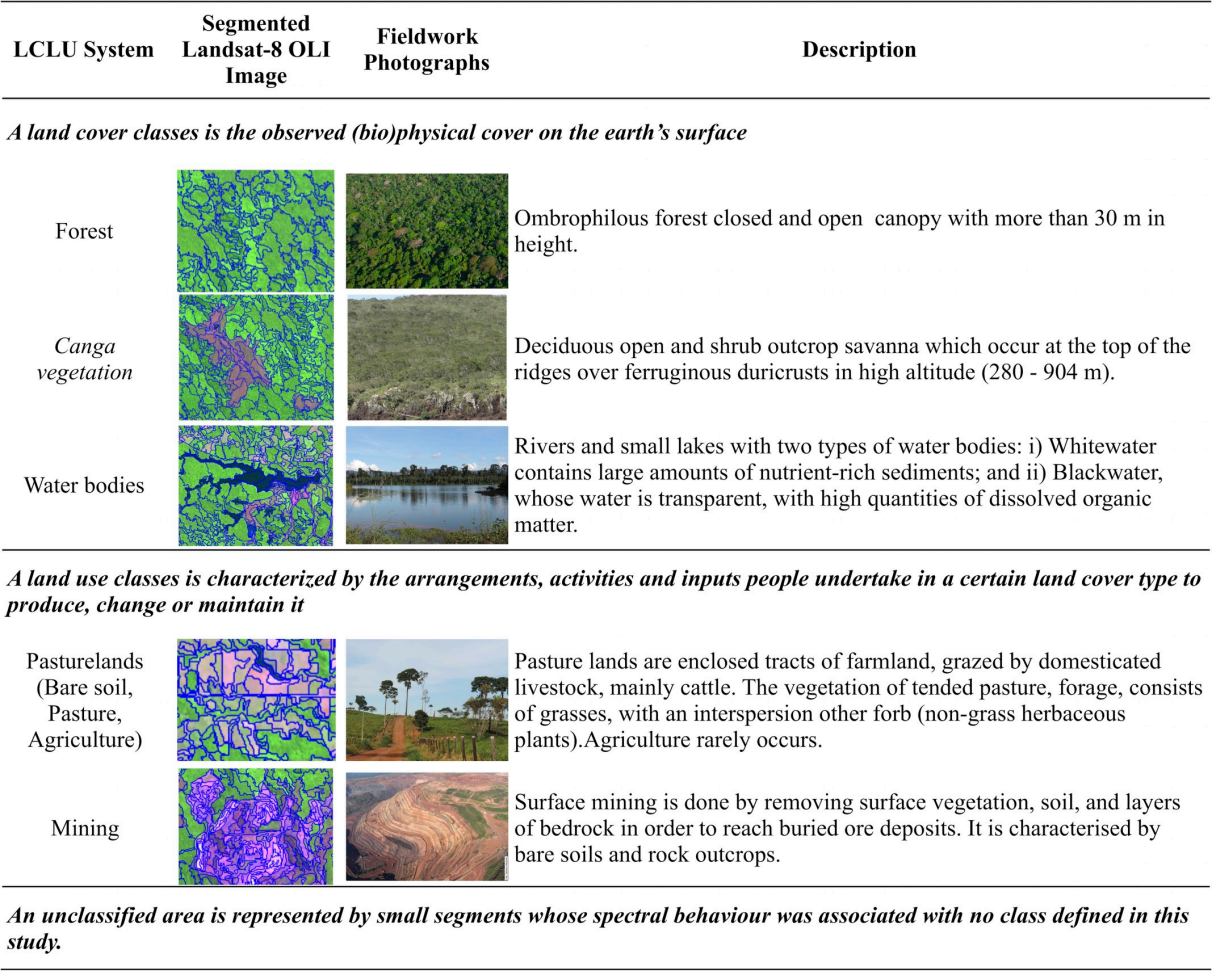
Description of the land-cover and land-use classification system used in this study. The Landsat images were obtained from USGS Earth Explorer (https://earthexplorer.usgs.gov). All photographs were taken by the authors and are copyright-free.

Fieldwork was conducted in 2014 and 2015 to determine the LCLU classes (e.g., canga, forest, and water) using panoramic digital photographs. During the fieldwork, 166 ground control points (GCPs) were collected using a differential global positioning system (DGPS) with reliable real-time positioning through the OmniSTAR mode for decimetre-level accuracy. These GCPs were used to validate the 2016 Landsat-8 OLI image classification. Training and validation samples were defined per class based on the GCPs. These data were also complemented by Google Earth Pro online high-resolution imagery. Regardless of the up to thirty-year difference among the images and the field data acquisition, all georeferenced field descriptions and photographs obtained in 2014 and 2015 matched the previous LCLU classes observed in the 1984 and 2001 Landsat-5 TM images. Fig 2 illustrates the classes recognized in the Landsat images and in the fieldwork, presenting a brief description of their characteristics.

### Measuring land-cover and land-use changes

**Fig 3:**
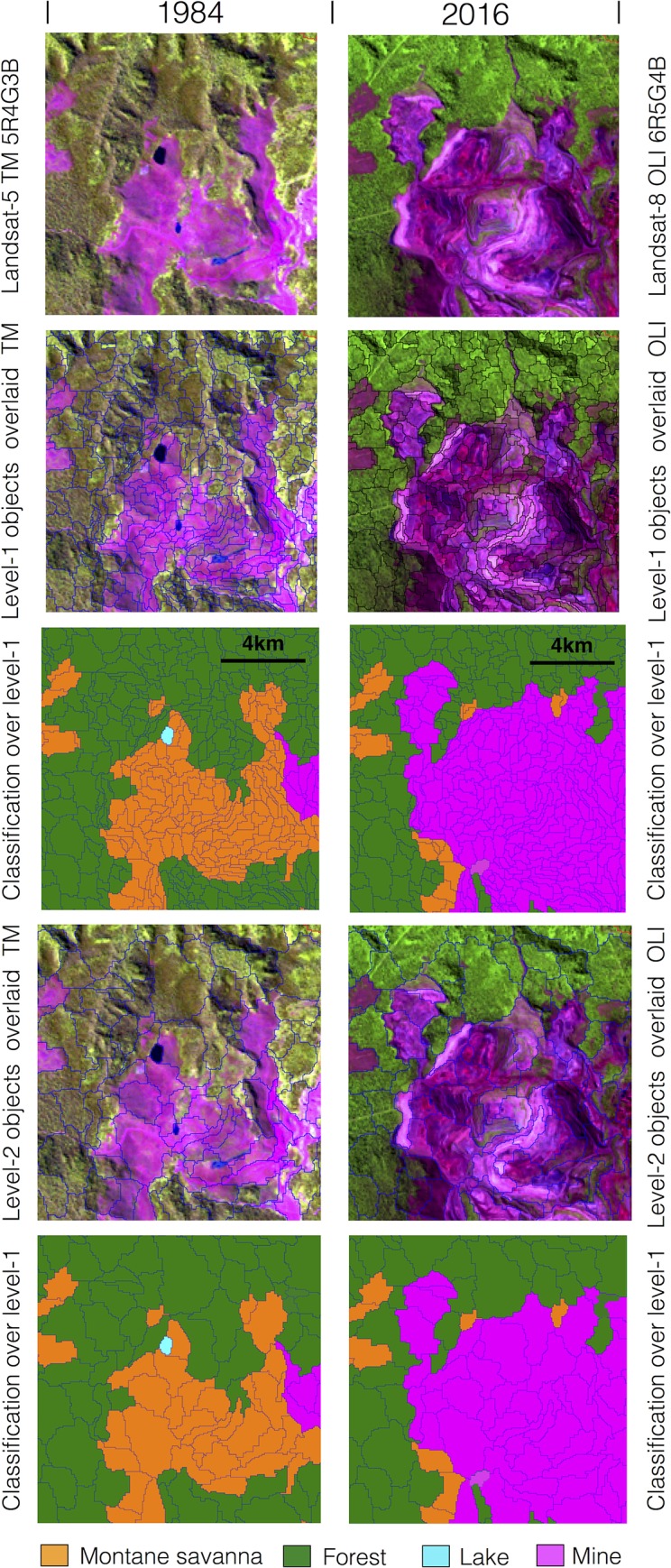
Example of segmentation and classification carried out from the 1984 Landsat-5 TM 5R4G3B and 2016 Landsat-8 OLI 6R5G4B images. Observed Landsat images are shown in colour composition; level 1 objects are overlaid on Landsat imagery; thematic classification for level 1 objects; level 2 objects are overlaid on imagery; and final result of thematic aggregation for level 2 objects. The Landsat images were obtained from USGS Earth Explorer (https://earthexplorer.usgs.gov). All other layers were produced by the authors and are copyright-free.

To estimate the canga area at the four snapshots in time, we used a multiresolution segmentation algorithm based on the homogeneity definition [34]. The three-date segmentation was conducted from all of the ground reflectance bands from the 1984 and 2001 Landsat-5 TM and 2016 Landsat-8 OLI images since they had the same spatial resolution (30 m). They were segmented using weight five for the near-infrared band and the NDVI index, and weight one for all other bands. The three-date segment was copied and used to classify the LCLU classes based on the 1973 Landsat-1 MSS, 1984 and 2001 Landsat-5 TM and 2016 Landsat-8 OLI images. This process followed geographic object-based image analysis (GEOBIA), combining the advantage of quality human interpretation and the capacities of quantitative computing [35]. For the purpose of detecting LCLU changes, we carried out a segmentation process from three separate single-date images, such as used by Desclée et al. [36] and Duveiller et al. [37]. Multi-date segmentation allowed for the comparison of three single images based on objects with the same geometry, delineating spatially and spectrally consistent segments and avoiding misclassification, allowing for the most accurate and rapid process and reducing additional processing efforts of outlining polygons [38]. All Landsat TM and OLI bands (ground reflectance values) of images (1984, 2001 and 2016) were used as input layers in the segmentation process. Fig 3 illustrates the step-by-step multi-date segmentation and classification process in a small mining site in the study area.

The segmentation approach was developed in two levels (Fig 3). Small objects were created in the level 1 segmentation (~1 ha), corresponding to approximately 9 Landsat TM and OLI pixels. Later, objects generated during the process of segmentation at level 1 were grouped to coarser objects in level 2, with a size of 4 ha, equivalent to 36 Landsat TM and OLI pixels. The segmentation in Level 2 aimed to reduce the number of objects and increase the size of polygons to facilitate the visual interpretation and change detection analysis. In regards to the definition of the scale parameter (*h*_*sc*_), several unsupervised and supervised methods are available to define the optimal scale parameter [39]. However, the selection of appropriate scale parameters is heavily dependent on trial-and-error exploration, which is iterative and time-consuming [40], because there is no obvious mathematical relationship between scale parameters and the success of the segmentation [39]. The segmentation shape (w_sp_), compactness (w_cp_) and scale (h_sc_) parameters were established as being equal to 0.1, 0.5, and 10, respectively, for all images. The h_sc_ parameters at level 1 and level 2 segmentation were 10 and 5, respectively, to image segmentation with minimum object sizes approximately 1 and 4 ha, respectively.

During the process of the automated classification of each image, we adopted membership functions to describe specific properties of the objects based on all Landsat spectral bands and elevation data obtained from the Shuttle Radar Topography Mission (SRTM). This process allowed for various features in the description of classes to be integrated by logical operators. The selection of features was assisted by an analysis of separability of the comparable classes. Each class was classified separately in the domain of the image object level using the filter class “unclassified”, according to the following order: i) forest; ii) water; iii) pasturelands; iv) mines; and v) canga vegetation. It is important to emphasize that the 1973 Landsat-1 MSS image contains i) the canga areas before the mining project, ii) the 1984 Landsat-5 TM image coinciding with the year of the Carajás Mining Project installation, iii) the 2001 Landsat-5 TM image representing the mid-term age of mining project, and iv) the 2016 Landsat-8 OLI image representing the current condition, when all iron ore exploitation projects (N4-N5, S11D and Serra Leste mines) were already in operation.

The LCLU changes were also analysed based on the “from-to” spatiotemporal change detection approach [35, 41] to recognize the trajectories of thematic classes from 1973–1984, 1984–2001, 2001–2016, and 1973–2016. We identified five classes that did not change over the period of investigation (forest–forest, savanna–savanna, lake–lake, mine–mine, and pasturelands–pasturelands) to understand their possible change trajectories, related to the conversions “from- to” of forests to pasturelands, forests to mines, canga to mines, canga to pasturelands, pasturelands to mines, and lakes to mines.

### Assessing the classification accuracy of LCLU classes

An object-based accuracy assessment is different from pixel-based validation due to the sampling units, i.e., objects vs. pixels [42]. However, a generally accepted approach is that classified polygons can be validated by GCPs [43]. To assess the classification accuracy of the 2016 Landsat-8 OLI image, 166 GCPs collected during fieldwork along accessible roads were used. As older GCPs and thematic maps were unavailable for the 1973, 1984 and 2001 Landsat-5 TM images, approximately 154, 159 and 137 validation points, respectively, were randomly stratified using the PCI Geomatica 2016 software. Hence, the accuracy of the Landsat image classifications was assessed using non-normalized and normalized confusion matrices [44]. The producer and user accuracies [45], Kappa per class, Kappa index of agreement and overall accuracy were also determined [43].

## Results

**Fig 4:**
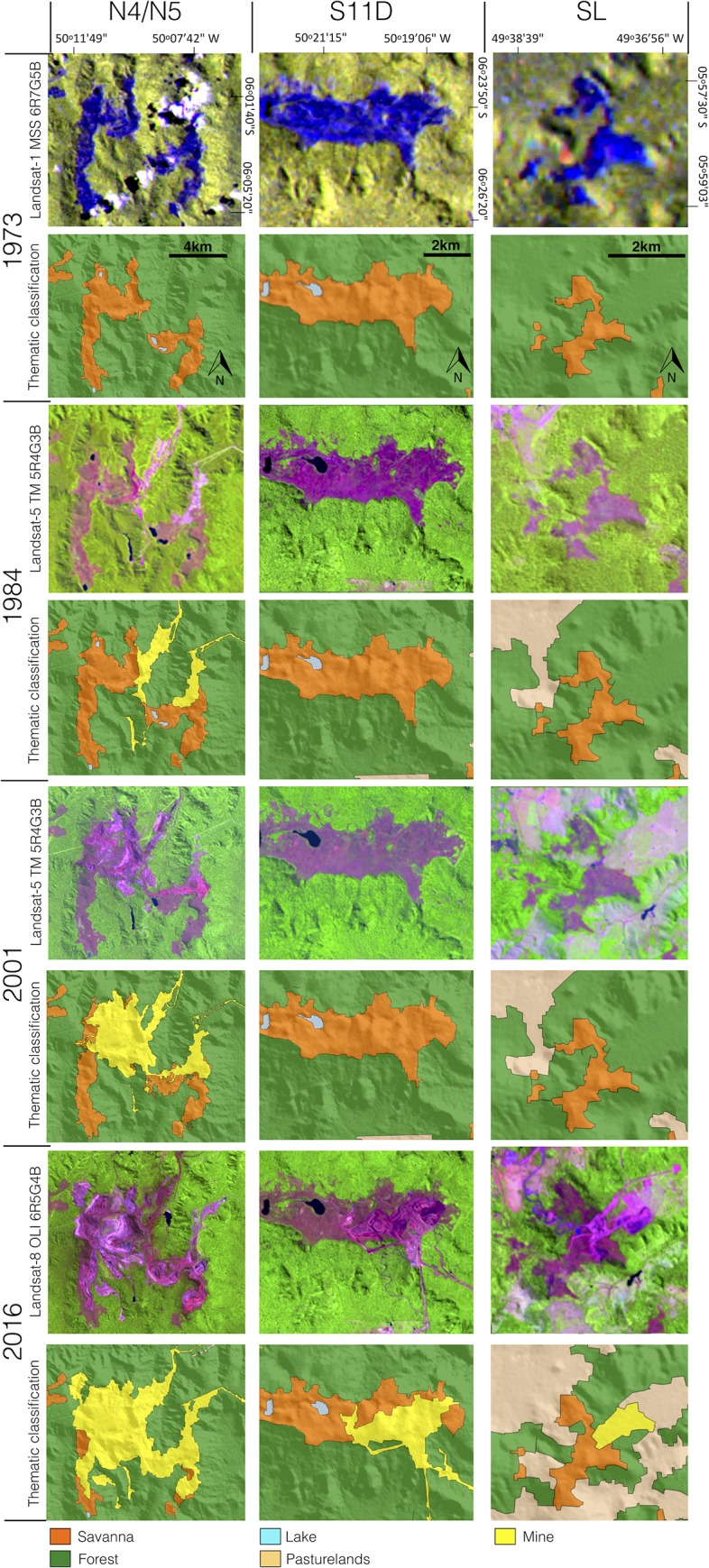
Landsat images, land cover (canga, forests and lakes) and land use (N4-N5, S11D and SL mines and pasturelands) maps from 1973, 1984, 2001 and 2016 in the Carajás Mineral Province. **See location of the mines in Fig 1.** The digital elevation model (SRTM, 1 arc-second) and Landsat images were obtained from USGS Earth Explorer (https://earthexplorer.usgs.gov). All other layers were produced by the authors and are copyright-free.

To assess the relationship between the Landsat image interpretations and terrain features, field campaigns were conducted in the study area to improve GEOBIA analysis, aiming to identify and map the different land cover and land use units. Fig 4 shows the replacement of canga vegetation observed in 1973 by open pit mines in 1984, 2001 and 2016 in the three sites exploited by the mining industry in the CMP (the N4-N5, SL and S11D mines).

**Fig 5:**
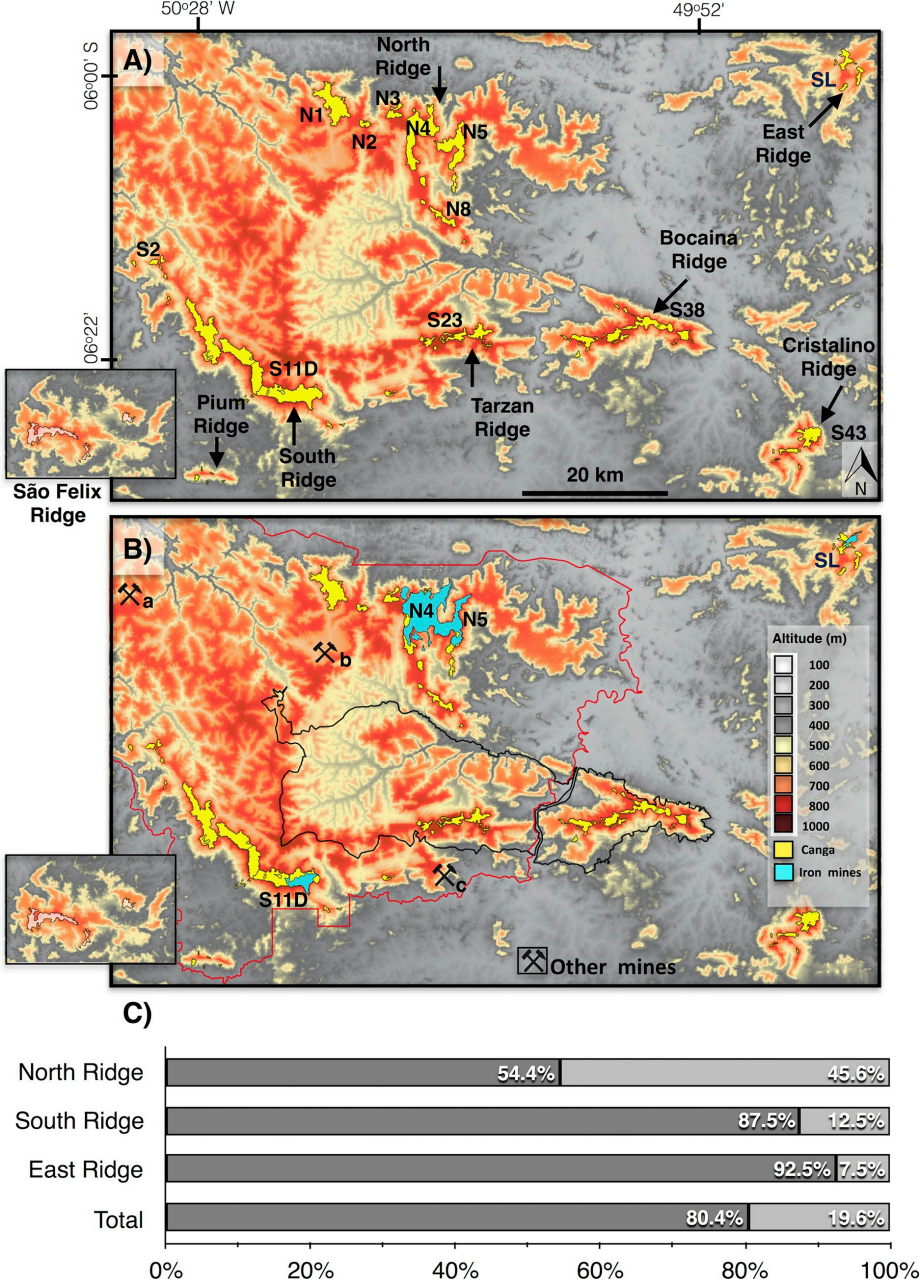
**Digital elevation model extracted from the Shuttle Radar Topography Mission (SRTM) showing canga areas (yellow polygons) (A) before implementation of mining projects and (B) currently, with mining areas (blue polygons).** The limits of the two protected areas are shown, the Carajás National Forest (red line) and the Campos Ferruginosos National Park (black line), created in 1998 and 2017, respectively. The São Felix Ridge is illustrated as a window, 160 km from the South Ridge. (C) The canga areas converted to mining structures between the 1970s and 2016 over the North, South and East Ridges, and over total canga area. Letters N, S and SL represent the geographic locations of North, South, and East ridges, respectively. Other mines: a = Igarapé Bahia, b = Azul, and c = Project 118. The digital elevation model (SRTM, 1 arc-second) was obtained from USGS Earth Explorer (https://earthexplorer.usgs.gov) and the National Forest and National Park shapefiles from ICMBIO (http://mapas.icmbio.gov.br/i3geo/datadownload.htm). All other layers were produced by the authors and are copyright-free.

The multiresolution classification based on the GEOBIA analysis effectively classified the canga vegetation and its related mines. Fig 5 shows these classes distributed within the study site throughout the years before mine implementation, indicating the pristine area of canga vegetation (Fig 5A), its current extent and the area of mining activities in 2016 (Fig 5B).

Based on random samples collected from the Landsat images, the results indicated that the overall accuracies and Kappa indices were 98.2 and 96.9 for the 2016 Landsat-8 OLI, 95.1 and 93.5 for the 2001 Landsat-5 TM, 95.8 and 94.4 for the 1984 Landsat-5 TM, and 95.6 and 93.6 for the 1973 Landsat-1 MSS images, respectively (S1 Table). The overall accuracies indicate that a large majority of segments were correctly identified according to the reference data (random samples). Some lake pixels were classified as artificial lakes in mines, while some mine samples were classified as forests (e.g., reclaimed areas colonized by grasslands) and canga vegetation, whose spectral responses are very similar to those of outcrops in mines. The highest omission errors occurred in lakes (18.2%) for the 2001 Landsat-5 TM image, while the highest commission errors occurred in the pasturelands class for the 1973 Landsat-1 MSS image (11.8%). The confusion between forests, pasturelands and mines can be explained by the regeneration of small patches of secondary forest in pasture areas and the revegetation of open pit mines with grasses. Misclassification of segments belonging to these classes can be observed in S1 Table.

The classification shows that canga vegetation in the CMP occupied an area of 144.2 km^2^ in 1973, before the implementation of the Carajás N4-N5 mines and open pit mining exploitation (Table 1, Fig 5A). From 1973 to 1984, 1.1 km^2^ of canga was suppressed in the Carajás North Ridge, increasing to 14.7 km^2^ in 2001 and to 22 km^2^ in 2016. In the South Ridge, where canga suppression began in 2014, 6 km^2^ of canga was removed. In the East Ridge, only 0.4 km^2^ of canga was suppressed from 2012 to 2016. Overall, the development of the mining projects in the Carajás region caused a reduction of 19.6% to the original canga area (Fig 5B). Table 1 presents the canga area before the implementation of mining projects (1973) and its extent in 1984, 2001 and currently (2016) in different geographical sites.

**Table 1:** Canga areas before the implementation of mining projects (1973) and currently (2016) in different geographical sites. See location of sites in Fig 1.

Ridges Sites	1973		1984		2001		2016		Remaining
	Km^2^	%	km^2^	%	km^2^	%	km^2^	%	%
North	48.2	33.4	47.1	32.9	33.5	25.9	26.2	22.6	54.4
South	48.1	33.4	48.1	33.6	48.1	37.2	42.1	36.3	87.5
East	4.6	3.2	4.6	3.2	4.6	3.5	4.2	3.7	92.5
Tarzan	8.2	5.7	8.2	5.8	8.2	6.4	8.2	7.1	100
Bocaina	15.7	10.9	15.7	11.0	15.7	12.1	15.7	13.6	100
Cristalino	7.1	4.9	7.1	5.0	7.1	5.5	7.1	6.1	100
São Felix	11.5	8.0	11.5	8.1	11.5	8.9	11.5	9.9	100
Pium	0.7	0.5	0.7	0.5	0.7	0.5	0.7	0.6	100
Total	144.2	100	143.1	100	129.5	100	115.9	100	80.4

Canga vegetation was converted to mines on the North, South and East ridges, but to different extents. On the North Ridge, where the N4 and N5 mines are located, 45.6% of the canga vegetation was lost between 1973 and 2016. On the South and East Ridges, where the S11D and SL mines are located, 13% and 8%, of the canga vegetation were lost, respectively. On the Tarzan, Bocaina, Cristalino, Pium and São Felix Ridges, the canga areas remained unchanged (Fig 5C).

Inside the CNF protected area, where the three largest iron ore mines are located, there was 105.2 km^2^ of canga vegetation before mining implementation, which represents 2.5% of the CNF area and 73% of the total area of canga vegetation in the Carajás region. The N4 (16.6 km^2^), S11D (16.2 km^2^), S11A (14.5 km^2^), N1 (12.1 km^2^) and N5 (11.8 km^2^) ridges contained the largest canga areas in the study site. Over the past three decades, mining activities suppressed 28.3 km^2^ of the canga area in the CMP, especially on the N4 (13.0 km^2^), N5 (8.9 km^2^), and S11D (6.0 km^2^) ridges within the CNF. This area represents 1.8% of the CNF protected area until July 2016. Outside of the CNF, there was 39 km^2^ of canga vegetation on the Bocaina, Cristalino, East and São Felix Ridges. The majority of this area (38.6 km^2^) remains conserved, and the suppression of 0.4 km^2^ of the canga area is associated with the implementation of the SL mine in the East Ridge (Fig 5). Based on the area of canga vegetation suppressed at each site (Table 1), the rates of suppression were calculated from the moment that mining activities began in 1984, 2012 and 2014 in N4-N5, SL and S11D mines, respectively. Hence, in the SL mine, the rate of canga suppression was approximately 0.1 km^2^.yr^-1^ from 2012 to 2016. In the S11D mine, the suppression rate reached 2 km^2^.yr^-1^ from 2013 to 2016, while in the N4-N5 mines, the rate decreased from 0.9 km^2^.yr^-1^ from 1984 to 2001 and to 0.5 km^2^.yr^-1^ from 2001 to 2016. S2 Table lists the names of the mines on each ridge, the land-cover type, the mean elevation and insertion in protected areas, and the canga area of each ridge in 1973, 1984, 2001 and 2016, and changes in the canga area of each ridge between 1973 and 2016.

**Fig 6:**
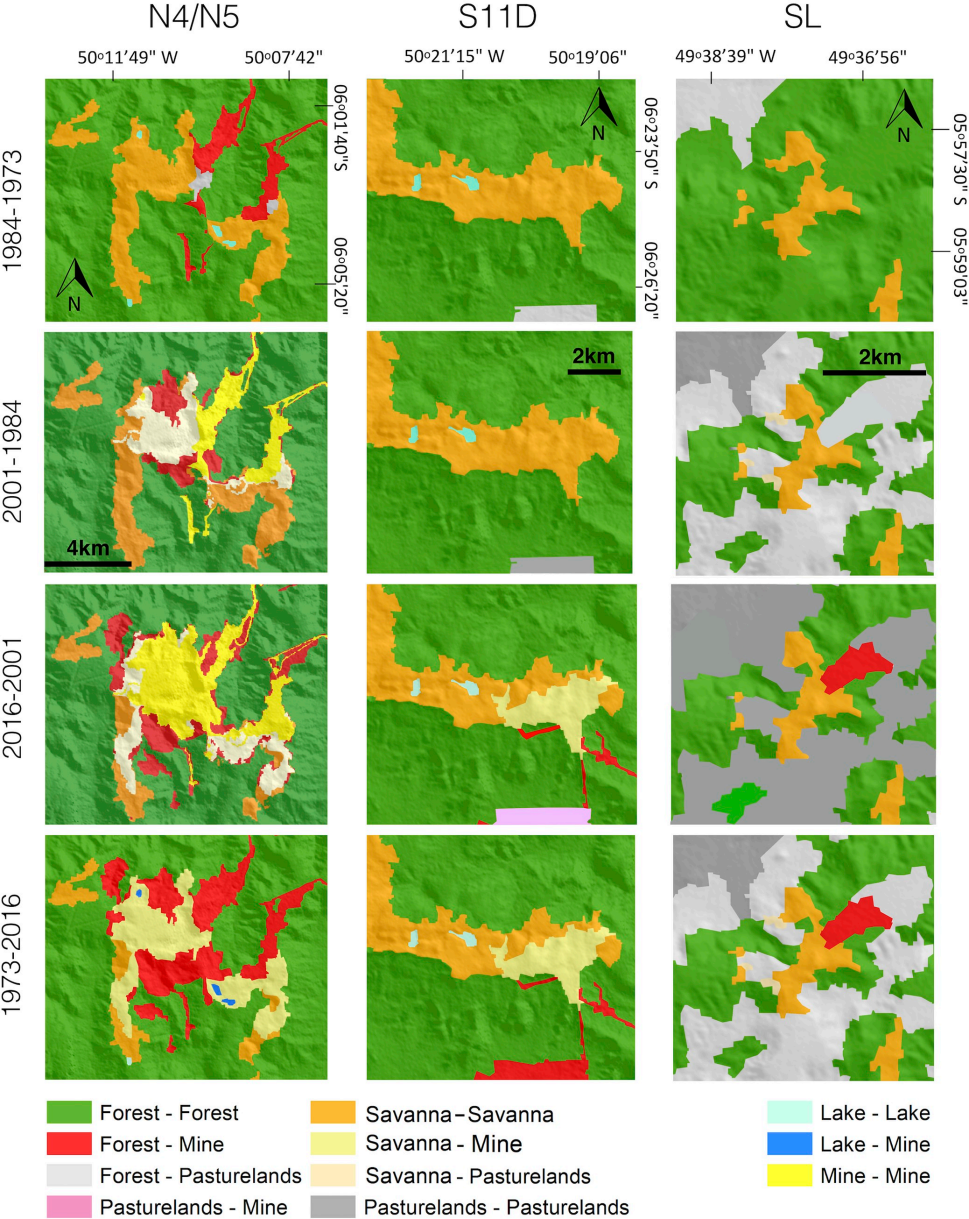
LCLU change detection maps between 1973–1984, 1984–2016, and 1973–2016 for the N4-N5, S11D and SL mines. The digital elevation model (SRTM, 1 arc-second) was obtained from USGS Earth Explorer (https://earthexplorer.usgs.gov) and the National Forest and National Park shapefiles from ICMBIO (http://mapas.icmbio.gov.br/i3geo/datadownload.htm). All other layers were produced by the authors and are copyright-free.

Fig 6 illustrates the LCLU changes based on a bi-temporal image analysis. The change between 1973 and 1984 indicates that unchanged forests were the largest class (248 km^2^). Forests were the main class converted to mines (8.6 km^2^), followed by the conversion of canga to mines (1.1 km^2^). Between 1984 and 2001, LCLU changes were most notable in the conversion from canga to mines (8.8 km^2^) and from forests to mines (5.7 km^2^). From 2001 to 2016, 15.4 km^2^ of canga were converted to mines and 10.6 km^2^ of forests were converted to mines in the N4-N5 mines, while 0.4 km^2^ and 6 km^2^ of canga were converted to mines in SL and S11D, respectively. During the entire period investigated (1973–2016), the main land cover changes were associated with conversions from forests to mines (26.6 km^2^) and canga to mines (24.3 km^2^). Table 2 shows all quantifications of LCLU changes between the periods of investigation.

**Table 2:** Quantification of land cover and land use changes between 1973–1984, 1984–2016, and 1973–2016 using the “from-to” object detection approach.

**a)**			
**1973 LCLU Class**	**1984 LCLU Class**	**Area (km**^**2**^**)**	**%**
Forest	Forest	248.18	79.90
	Mine	8.57	2.76
	Pasturelands	4.56	1.47
Lake	Lake	0.89	0.29
Canga	Forest	0.02	0.01
	Mine	1.08	0.35
	Canga	47.32	15.23
**Total**		310.61	100.00
**b)**	** **		
**1984 LCLU Class**	**2001 LCLU Class**	**Area (km**^**2**^**)**	**%**
Forest	Forest	239.51	77.11
	Mine	5.73	1.84
	Pasturelands	14.00	4.51
Lake	Lake	0.67	0.22
	Mine	0.24	0.08
Mine	Mine	9.31	3.00
Canga	Mine	8.85	2.85
	Canga	23.71	7.63
Pasturelands	Pasturelands	8.58	2.76
**Total**		310.61	100.00
**c)**			
**2001 LCLU Class**	**2016 LCLU Class**	**Area (km**^**2**^**)**	**%**
Forest	Forest	219.53	70.68
	Mine	10.58	3.41
	Pasturelands	5.26	1.69
Lake	Lake	0.52	0.17
	Mine	0.12	0.04
Mine	Mine	22.00	7.08
Canga	Mine	15.40	4.96
	Canga	20.71	6.67
Pasturelands	Mine	1.78	0.57
	Pasturelands	14.70	4.73
**Total**		310.61	100.00
**d)**			
**1973 LCLU Class**	**2016 LCLU Class**	**Area (km**^**2**^**)**	**%**
Forest	Forest	218.80	70.44
	Mine	26.58	8.56
	Pasturelands	13.36	4.30
Lake	Lake	0.52	0.17
	Mine	0.36	0.11
Canga	Mine	24.30	7.82
	Canga	23.88	7.69
	Pasturelands	0.23	0.07
Pasturelands	Pasturelands	2.58	0.83
**Total**		310.61	100.00

## Discussion

In this paper, we mapped and quantified canga vegetation as well as the changes in LCLU classes in the Carajás region, mainly focusing on the effects of mining operations. Previously, the canga area in Brazil was noted as being approximately 261.6 km^2^, with 102 km^2^ in the Iron Quadrangle and 103 km^2^ in the Carajás region [23]; however, the methods used for these estimates were not fully described. Our results show that the Carajás region originally included 144.2 km^2^ of canga vegetation, a figure 40% higher than the previous estimates [23]. As mentioned before, previous studies do not describe how canga area was estimated. It is probable that canga area was calculated from analogic aerial photographs, whose spatial distortion was not corrected.

Mining activities suppressed 28.3 km^2^ of canga (19.6%), while large areas of canga were still conserved in the Carajás region (115.9 km^2^). This area corresponds to 80.4% of the pristine canga area and represents one of the largest conserved canga ecosystem areas in Brazil. In the Iron Quadrangle (Minas Gerais State), the total area covered by canga vegetation is approximately 100 km^2^ [46]. However, this estimated extent must be reviewed due to the methods used to estimate the area, based on uncorrected analogic aerial photographs. According to Sonter et al. [10], 17.6 km^2^ of canga area has been cleared by mining activities. The percentage of canga suppression in the Iron Quadrangle (approximately 17%) is similar to that in the Carajás region.

The high rates of canga vegetation suppression observed in the S11D mine are associated with early stages of mine implementation. According to the S11D project, the useful lifespan of a mine is approximately 30 years (http://www.vale.com/en/initiatives/innovation/s11d/pages/default.aspx). Hence, the canga vegetation suppression observed over the past three years (~ 6 km^2^) will increase less than 10% over the next 30 years, and the suppression rate will reach approximately 0.2 km^2^.yr^-1^. In the N4-N5 mines, the main rump-up period is represented by the changes from 1984 to 2001, where canga suppression is almost twice as high as in the open pit phase from 2001 to 2016. However, if an increasing demand for iron ore occurs over the next several years, the useful lifespan of the mine will decrease, and the rates of canga vegetation suppression may increase. A similar process has been observed in terms of Amazon rainforest losses due to the large demands relative to Brazil’s natural resources, including land, timber, minerals and hydroelectric potential, demands mainly driven by the high prices of commodities [47, 48]. The results of Sonter et al. [49] supported a hypothesis that global demand for steel drives extensive land-use changes in the Iron Quadrangle, where increased steel production was correlated with increased iron ore production and mine expansion. Consequently, this process is also responsible for increasing charcoal production and the expansion of subsistence crops. In a future study, this hypothesis will be evaluated in relation to Carajás mining projects.

The accurate mapping of areas is vital to guiding conservation strategies, especially for canga vegetation in the eastern Brazilian Amazon where iron ores are located. Many authors have already described the threats to canga and the challenges of conserving this vegetation [16, 17, 50, 51]. Among the threats, vegetation suppression for open pit mining, fire and invasive plant species have been noted as the major threats [17]. The greatest challenge of conserving canga vegetation is to manage biological invasions and create protected areas to avoid species losses [23, 51].

The canga areas in the Carajás region are partially within the CNF (67%). This category of protected area allows for sustainable use, including sustainable mining activities. The CNF has also contributed to forest conservation, with surveillance improving the ability to inspect different anthropogenic impacts associated with fire, human settlements and gold digging. Otherwise, other economic activities such as livestock and agriculture would have threatened the natural land cover, mainly of tropical rainforests, which would have been completely converted to pasturelands or croplands as observed in adjacent areas [21]. To improve the conservation of canga vegetation in this area, the Brazilian Institute for Biodiversity Conservation (ICMBio) created the Campos Ferruginosos National Park (CFNP) in June 2017, an integral conservation unit financed by offset strategies for mining operations. This park includes the Bocaina and Tarzan Ridges, totalling approximately 24 km^2^ of canga vegetation that represent 21% of canga area in the CMP, where mining activities will not be allowed. Hence, an additional step was taken towards canga vegetation conservation in the Amazon region. In addition, the recently-published CNF Management Plan defines the areas to be protected and those that can be mined and divides these areas into categories, which include i) preservation areas, with 15% of the total area of the CNF where human activities are not allowed; ii) transition areas, covering 15% of the CNF with mixed areas of conservation and management; iii) mining areas where mining activities can be conducted, which is 14% of the CNF area; iv) forest areas designated for sustainable management (50% of the CNF area); and v) special and public use areas, covering 6% and designed for infrastructure and general use of the CNF [52]. The areas of canga vegetation destined for mining consist of mines (see location in Fig 4) that are already installed, such as N4 and N5 on the North Ridge, Azul, Igarapé Bahia, Project 118, and S11D on the South Ridge, among others that will be installed in the near future (N1, N2, N3 in the North Ridge).

The Carajás ridges are a clear example of challenges to the conservation and exploitation of natural resources. The growing demands of society and the quality of the Carajás iron ore have encouraged the exploitation of this resource in these areas. However, for sustainable mining, adequate conservation strategies need to be implemented properly, and scientific research is a key aspect of this process. Heavy-metal pollution in water from iron ore exploitation has not yet been detected in the Carajás region [53].The proper determination of areas outside the Carajás region to be protected as offsets may represent an important tool for the protection of species in the region.

## Conclusion

Remote sensing data and GIS tools provided four snapshots in time, permitting the mapping of canga areas and the quantifying of changes in land use. Based on the image analysis, we observed that canga areas in the CMP are 40% higher than previous estimates. The suppression of canga vegetation was associated with the implementation of mining projects, which favours the suppression of forest areas for canga conservation. It is important to emphasize that the most substantial vegetation suppression occurred during the earlier stages of mine implementation, from 1984 to 2001. Later, vegetation suppression was substantially reduced during the open pit mining phase from 2001 to 2016, where a hole is excavated from the earth’s surface. After three decades of mineral exploitation, 80.6% of the canga area in the Carajás region remains untouched. Government and mining industries have used offsets to compensate the unavoidable impacts of iron ore exploitation. Hence, the CFNP was created to protect 21% of the canga area in the CMP. We believe that mapping and quantifying the areas of canga vegetation that have already been lost can be considered the first step towards conserving this important rocky environment.

## Supporting information

S1 Table **Confusion matrix of the GEOBIA of the 2016 Landsat-8 OLI (A), 2001 Landsat-5 TM (B), 1984 Landsat-5 TM, (C) and 1973 Landsat-1 MSS (D) classifications.** The matrix shows the number of sample points, omission and commission errors, user and producer accuracies, Kappa index per class, overall accuracy and general Kappa index.
(XLSX)

S2 Table General characteristics of canga ridges in the study site. CNF = Carajás National Forest, CFNP = Campos Ferruginosos National Park.
(XLSX)
